# Supplementary material for: Detection of NTRK Fusions and TRK Expression and Performance of pan-TRK Immunohistochemistry in Routine Diagnostics: Results from a Nationwide Community-Based Cohort
Source: Diagnostics (Basel). 2022 Mar 9;12(3):668. doi: 10.3390/diagnostics12030668 (PMC8946871; doi:10.3390/diagnostics12030668)
Supplement: Supplementary file 1 [file diagnostics-12-00668-s001.zip › diagnostics-1620692-supplementary.pdf]

SUPPLEMENTARY INFORMATION

Detection of *NTRK* fusions and TRK expression and performance of pan-TRK immunohistochemistry in routine diagnostics: results from a nationwide community-based cohort

Bart Koopman, MD, Chantal C. H. J. Kuijpers, PhD, Harry J. M. Groen, MD, PhD, Wim Timens, MD, PhD, Ed Schuuring, PhD, Stefan M. Willems, MD, PhD, Léon C. van Kempen, PhD

Table of Contents

Supplementary Figures 2

Figure S1. Patient selection procedure 2

Figure S2. Interpretation of TRK testing results in accordance with various (inter)national guidelines 3

Supplementary Tables 4

Table S1. List of patients with a molecular-confirmed *NTRK1–3* fusion (*n*=69) 4

Supplementary Figures

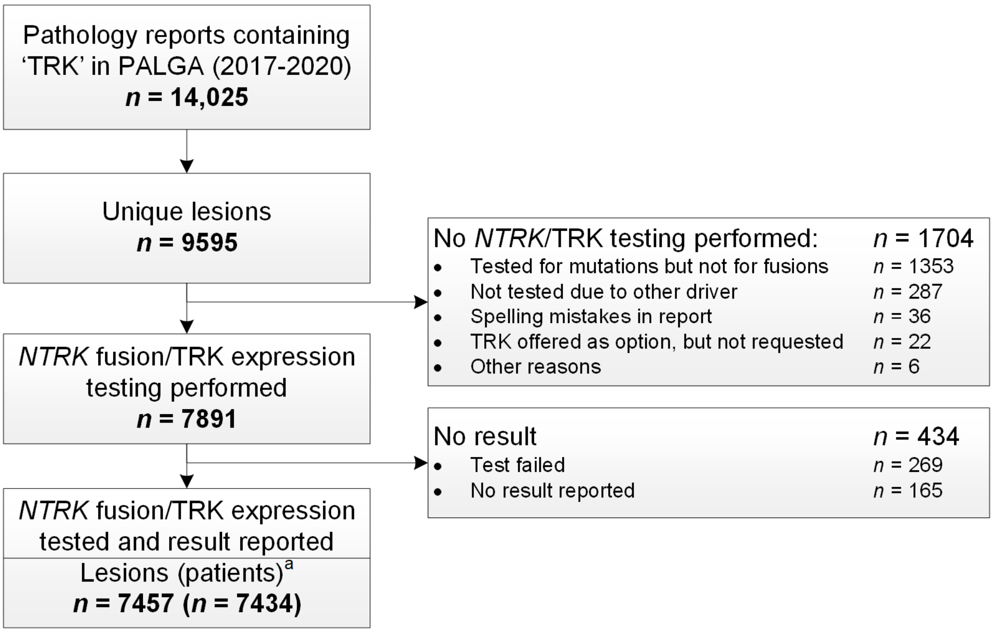

Figure S1. Patient selection procedure

Flow chart depicting patient selection. All pathology reports in the Netherlands that contained the search term 'TRK' between 2017–2020 were extracted from PALGA. Only tumors tested for TRK expression and/or the presence of an *NTRK* fusion were included. Patients were excluded if the test yielded no result, or if the result was not reported.

[a]23 patients had two unique tumors that were both tested for TRK expression and/or *NTRK* fusions

PALGA, The nationwide network and registry of histo- and cytopathology in the Netherlands.

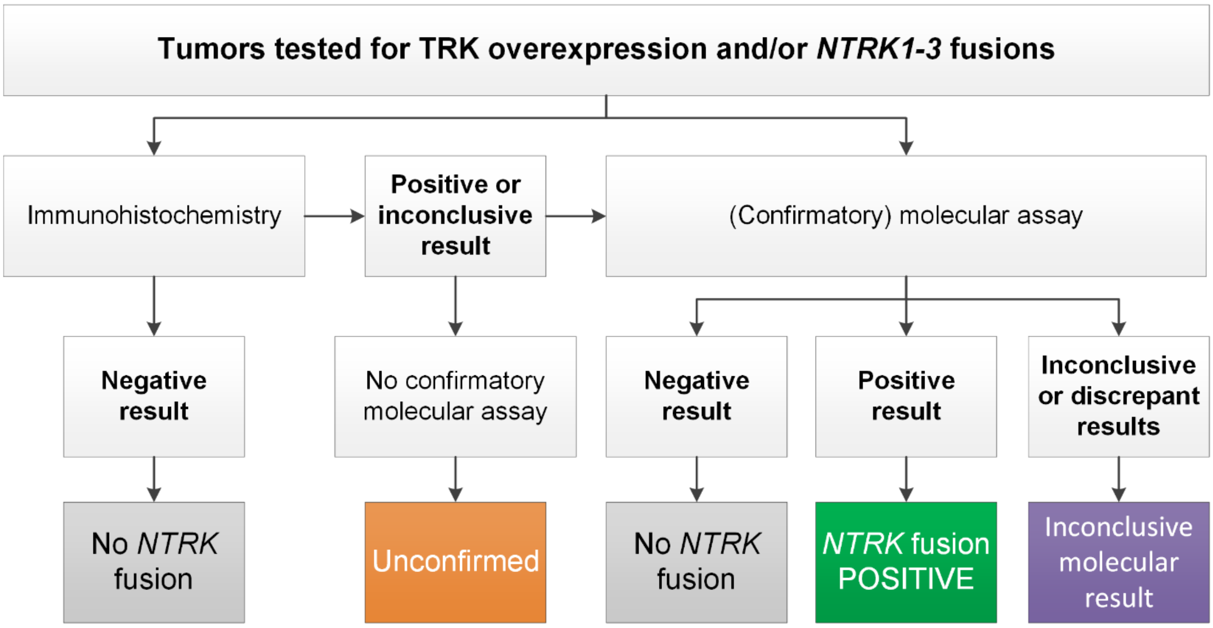

Figure S2. Interpretation of TRK testing results in accordance with various (inter)national guidelines

In accordance with (inter)national consensus guidelines,[20–23] testing results were interpreted as "NTRK fusion positive" only if a molecular assay unequivocally demonstrated the presence of an *NTRK* fusion. The test result was interpreted as "unconfirmed" if the positive or inconclusive IHC result was not confirmed with a molecular assay. If the molecular assay results were inconclusive, or if multiple molecular assays demonstrated discrepant results, the result was interpreted as "inconclusive".

Supplementary Tables

Table S1. List of patients with a molecular-confirmed *NTRK1–3* fusion (*n*=69)

ID	Sex	Age	Year	Diagnosis	Other drivers[a]	Test category	TRK fusion	IHC antibody	Result	Molecular assay	Result
				**Lung cancer (*n*=9)**							
1075	F	50	2017	Lung adenocarcinoma	No (F/M)	RNA	*SYNJ1-NTRK3*	–	–	Archer FP CTL	POSITIVE
2890	F	51	2017	Lung adenocarcinoma	*EGFR*[b]	RNA	*PRG4-NTRK1*	–	–	Archer FP CTL	POSITIVE
1303	M	73	2019	Lung adenocarcinoma	No (F/M)	RNA	*ETV6-NTRK3*	–	–	Archer FP CTL	POSITIVE
3900	M	73	2020	Lung adenocarcinoma	No (F/M)	RNA	*GRIPAP1-NTRK1*	–	–	Archer FP Lung	POSITIVE
6326	M	72	2020	Lung adenocarcinoma	No (F/M)	IHC+RNA	*TPM3-NTRK1*	Unspecified	POSITIVE	Multiplex RNA analysis	POSITIVE
7145	F	37	2020	Non-small cell lung cancer, NOS	*EGFR*[c]	IHC+RNA	*EFNA1-NTRK1*	Unspecified	POSITIVE	Archer FP Lung	POSITIVE
8986	F	37	2020	Lung adenocarcinoma	No (F/M)	IHC+FISH+RNA	*TRIM24-NTRK2*	EPR17341	POSITIVE	NTRK1/3 FISH (NOS)<br>Archer FP Lung	POSITIVE
9220	F	62	2020	Non-small cell lung cancer, NOS	*KRAS*[d]	IHC+RNA	*CD74-NTRK1*	Unspecified	POSITIVE	NanoString (custom)	POSITIVE
9291	M	49	2020	Lung adenocarcinoma	No (F/M)	IHC+RNA	*ETV6-NTRK3*	Unspecified	POSITIVE	Archer FP Solid Tumor	POSITIVE
				**Soft tissue/bone tumor (*n*=16)**							
714	F	45	2017	Chondrosarcoma	No (F)	IHC+RNA	*CHD2-NTRK3*	Unspecified	Negative	Archer FP CTL	POSITIVE
3618	M	52	2019	*NTRK*-rearranged spindle cell neoplasm	No (F)	IHC+RNA	*TPM3-NTRK1*	Unspecified	POSITIVE	Archer FP CTL	POSITIVE
3677	F	9	2019	Infantile fibrosarcoma	No (F/M)	IHC+FISH+RNA	*EML4-NTRK3*	Unspecified	POSITIVE	NTRK3 FISH (ZytoLight)<br>Archer FP Sarcoma v1<br>Unspecified molecular test	POSITIVE
5684	F	58	2019	Solitary fibrous tumor	No (F)	IHC+RNA	*NTRK1* fusion, NOS	Unspecified	Negative	NanoString (custom)	POSITIVE
6021	M	40	2019	Angiofibroma	No (F)	RNA	*STRN-NTRK2*	–	–	Archer FP (custom)	POSITIVE
5275	F	27	2019	Solitary fibrous tumor	No (F/M)	RNA	*UBE2F-NTRK3*	–	–	Archer FP Sarcoma v1	POSITIVE
5326	M	2	2019	Infantile fibrosarcoma	No (F)	RNA	*TPR-NTRK1*	–	–	Archer FP Lung	POSITIVE
4752	F	30	2020	Adult fibrosarcoma	No (F/M)	RNA	*NTRK3* fusion, NOS	–	–	NanoString (custom)	POSITIVE
5229	M	9	2020	*NTRK*-rearranged spindle cell neoplasm	No (F)	IHC+FISH+RNA	*LMNA-NTRK1*	Unspecified	POSITIVE	NTRK1/2/3 FISH (ZytoLight)<br>NanoString (custom)<br>Unspecified molecular test	POSITIVE
5366	F	22	2020	*NTRK*-rearranged spindle cell neoplasm	No (F)	RNA	*TPM3-NTRK1*	–	–	NanoString (custom)	POSITIVE
5385	M	30	2020	*NTRK*-rearranged spindle cell neoplasm	No (F/M)	IHC+RNA	*CNTN4-NTRK3*	Unspecified	POSITIVE	Archer FP Sarcoma v1	POSITIVE
8934	M	16	2020	*NTRK*-rearranged sarcoma	No (F)	IHC+FISH+RNA	*TPM3-NTRK1*	Unspecified	POSITIVE	NTRK1 FISH (ZytoLight)<br>Multiplex RNA analysis	POSITIVE
8954	F	26	2020	*NTRK*-rearranged spindle cell neoplasm	No (F)	RNA	*MYH9-NTRK3*	–	–	Archer FP Sarcoma v1	POSITIVE
9032	F	16	2020	*NTRK*-rearranged sarcoma	No (F/M)	IHC+RNA	*TPM3-NTRK1*	Unspecified	POSITIVE	Archer FP Sarcoma v1<br>Archer FP Solid Tumor	POSITIVE
9262	F	11	2020	Lipofibromatosis-like neural tumor	No (F)	IHC+RNA	*TPR-NTRK1*	Unspecified	POSITIVE	Multiplex RNA analysis	POSITIVE
9494	M	0	2020	*NTRK*-rearranged spindle cell neoplasm	No (F)	FISH+RNA	*TPM3-NTRK1*	–	–	NTRK1/2/3 FISH (ZytoLight)<br>Archer FP (custom)	POSITIVE
				**Melanocytic tumor (*n*=10)**							
3262	F	4	2017	Melanoma arising in blue nevus	No (F/M)	IHC+RNA	*TPM3-NTRK1*	EPR17341	Negative	Archer FP CTL	POSITIVE

ID	Sex	Age	Year	Diagnosis	Other drivers[a]	Test category	TRK fusion	IHC antibody	Result	Molecular assay	Result
5555	F	33	2019	STUMP	No (F)	RNA	*LMNA-NTRK1*	–	–	NanoString (custom)	POSITIVE
6188	F	21	2019	Atypical/benign Spitz nevus	No (F)	IHC+FISH+RNA	*LMNA-NTRK1*	Unspecified	POSITIVE	NTRK1/2/3 FISH (ZytoLight)<br>Archer (NOS)	POSITIVE
6567	M	39	2020	Atypical/benign Spitz nevus	No (F/M)	IHC+RNA	*SQSTM1-NTRK2*	Unspecified	POSITIVE	Archer FP CTL	POSITIVE
9116	M	15	2020	Atypical/benign Spitz nevus	No (F/M)	IHC+RNA	*MYO5A-NTRK3*	Unspecified	Negative	Archer FP Lung	POSITIVE
8891	F	21	2020	Atypical/benign Spitz nevus	No (F)	IHC+RNA	*ETV6-NTRK3*	Unspecified	Inconclusive	Archer FP CTL	POSITIVE
9334	M	4	2020	Atypical/benign Spitz nevus	No (F/M)	IHC+RNA	*LMNA-NTRK1*	Unspecified	POSITIVE	NanoString (custom)	POSITIVE
9357	F	5	2020	Atypical/benign Spitz nevus	No (F/M)	IHC+RNA	*MYO5A-NTRK3*	EPR17341	Inconclusive	Archer FP (custom)	POSITIVE
9526	F	22	2020	Atypical/benign Spitz nevus	No (F/M)	RNA	*MYO5A-NTRK3*	–	–	Archer FP Lung	POSITIVE
9548	M	48	2020	Atypical/benign Spitz nevus	No (F/M)	IHC+RNA	*MYO5A-NTRK3*	Unspecified	Negative	Archer FP CTL	POSITIVE
				**Thyroid tumor (*n*=13)**							
671	F	79	2017	Papillary thyroid carcinoma	–	Unspecified test	*ETV6-NTRK3*	Unknown	Unknown	Unknown	POSITIVE
1316	M	79	2017	Hürthle cell carcinoma	No (F/M)	RNA	*ETV6-NTRK3*	–	–	Archer FP CTL	POSITIVE
3281	F	19	2017	Papillary thyroid carcinoma	No (F/M)	RNA	*ETV6-NTRK3*	–	–	Archer FP CTL	POSITIVE
3473	M	0	2018	Thyroid cancer, NOS	–	Unspecified test	*EML4-NTRK3*	Unknown	Unknown	Unknown	POSITIVE
1968	M	34	2019	Follicular variant of PTC	No (F/M)	RNA	*ETV6-NTRK3*	–	–	Archer FP CTL	POSITIVE
3402	F	15	2019	Papillary thyroid carcinoma	No (F)	RNA	*ETV6-NTRK3*	–	–	Archer FP CTL	POSITIVE
4854	M	55	2019	Follicular thyroid carcinoma	–	FISH	*NTRK1* fusion, NOS	–	–	NTRK1/2/3 FISH (ZytoLight)	POSITIVE
5880	F	54	2019	Follicular variant of PTC	–	IHC+FISH+RNA	*NTRK1* fusion, NOS	EPR17341	Inconclusive	NTRK1/2/3 FISH (ZytoLight)<br>Archer FP Lung (failed)	POSITIVE
5160	F	24	2019	Papillary thyroid carcinoma	No (F/M)	RNA	*ETV6-NTRK3*	–	–	Archer FP CTL	POSITIVE
4495	F	52	2020	Papillary thyroid carcinoma	–	IHC+FISH+RNA	*NTRK3* fusion, NOS	EPR17341	POSITIVE	NTRK1/2/3 FISH (ZytoLight)<br>Archer FP (custom) (failed)	POSITIVE
4584	F	53	2020	Poorly differentiated thyroid carcinoma	No (M)	IHC+FISH+RNA	*NTRK1* fusion, NOS	EPR17341	POSITIVE	NTRK1/2/3 FISH (ZytoLight)<br>Archer FP Lung (failed)	POSITIVE
5021	M	69	2020	Thyroid cancer, NOS	–	IHC+unspecified	*ETV6-NTRK3*	Unspecified	POSITIVE	Unknown	POSITIVE
9418	F	7	2020	Papillary thyroid carcinoma	No (F)	RNA	*ETV6-NTRK3*	–	–	Multiplex RNA analysis	POSITIVE
				**Salivary gland tumor (*n*=13)**							
2723	F	84	2017	Salivary gland secretory carcinoma	–	RNA	*ETV6-NTRK3*	–	–	RT-PCR (ETV6-NTRK3)	POSITIVE
2768	F	24	2017	Salivary gland secretory carcinoma	–	RNA	*ETV6-NTRK3*	–	–	RT-PCR (ETV6-NTRK3)	POSITIVE
3370	M	55	2017	Salivary gland secretory carcinoma	–	RNA	*ETV6-NTRK3*	–	–	RT-PCR (ETV6-NTRK3)	POSITIVE
384	F	55	2018	Salivary gland secretory carcinoma	–	RNA	*ETV6-NTRK3*	–	–	RT-PCR (ETV6-NTRK3)	POSITIVE
856	M	61	2018	Salivary gland secretory carcinoma	–	RNA	*ETV6-NTRK3*	–	–	RT-PCR (ETV6-NTRK3)	POSITIVE
1534	F	71	2018	Salivary gland secretory carcinoma	No (F)	RNA	*ETV6-NTRK3*	–	–	RT-PCR (ETV6-NTRK3)	POSITIVE
1568	F	55	2018	Salivary gland secretory carcinoma	–	RNA	*ETV6-NTRK3*	–	–	RT-PCR (ETV6-NTRK3)	POSITIVE
2555	M	75	2018	Salivary gland secretory carcinoma	No (F/M)	RNA	*ETV6-NTRK3*	–	–	Archer FP Solid Tumor	POSITIVE
2634	M	54	2018	Salivary gland secretory carcinoma	–	RNA	*ETV6-NTRK3*	–	–	RT-PCR (ETV6-NTRK3)	POSITIVE
1574	M	59	2019	Salivary gland secretory carcinoma	–	RNA	*ETV6-NTRK3*	–	–	RT-PCR (ETV6-NTRK3)	POSITIVE
141	F	26	2019	Salivary gland secretory carcinoma	–	RNA	*ETV6-NTRK3*	–	–	RT-PCR (ETV6-NTRK3)	POSITIVE
638	M	71	2019	Salivary gland secretory carcinoma	No (F)	IHC+RNA	*ETV6-NTRK3*	Unspecified	Negative	Archer FP Solid Tumor	POSITIVE
7427	M	42	2020	Salivary gland secretory carcinoma	No (F)	RNA	*ETV6-NTRK3*	–	–	Archer FP (custom)	POSITIVE

ID	Sex	Age	Year	Diagnosis	Other drivers[a]	Test category	TRK fusion	IHC antibody	Result	Molecular assay	Result
				**Brain tumor (*n*=4)**							
5277	F	7	2019	Pleomorphic xanthoastrocytoma	No (F/M)	RNA	*NACC2-NTRK2*	–	–	Multiplex RNA analysis	POSITIVE
6239	F	0	2020	Infantile hemispheric glioma	No (F/M)	RNA	*ETV6-NTRK3*	–	–	Archer FP Lung	POSITIVE
8860	M	38	2020	Pilocytic astrocytoma	No (F)	RNA	*FMN2-NTRK2*	–	–	Archer FP Lung	POSITIVE
9268	M	6	2020	Diffuse low-grade glioma	No (F/M)	RNA	*BEND5-NTRK2*	–	–	Multiplex RNA analysis	POSITIVE
				**Breast cancer (*n*=2)**							
3561	M	28	2018	Breast secretory carcinoma	No (F)	RNA	*ETV6-NTRK3*	–	–	Archer FP Solid Tumor	POSITIVE
8361	F	71	2020	Breast secretory carcinoma	–	IHC+FISH	*ETV6-NTRK3*	Unspecified	POSITIVE	ETV6 FISH	POSITIVE
				**Cancer of unknown primary (CUP)**							
1185	M	69	2019	Non-small cell CUP	No (F/M)	IHC+FISH+RNA	*GP2-NTRK1*	Unspecified	POSITIVE	NTRK1 FISH (NOS)<br>Archer FP CTL	POSITIVE
				**Congenital mesoblastic neprhoma**							
5609	M	0	2019	Congenital mesoblastic nephroma	No (F)	FISH+RNA	*ETV6-NTRK3*	–	–	NTRK1/2/3 (ZytoLight)<br>ETV6 FISH<br>Multiplex RNA analysis	POSITIVE

– indicates the variable was not tested

[a]Other driver mutations or fusions detected through next-generation sequencing or multiplex RNA analysis. “No (F/M)” indicates no other mutations/fusions were found; “No (F)” indicates no other fusions were found with multiplex RNA analysis, but NGS was not performed; “No (M)” indicates no other mutations were found, but multiplex RNA analysis was not performed and a dash indicates the patient was not tested for other drivers (or that this was not specified).

[b]co-occurence of *EGFR* mutation at primary diagnosis (untreated)

[c]co-occurence of *EGFR* mutation, *NTRK* fusion acquired at resistance to EGFR-TKI

[d]co-occurrence of *KRAS* mutation at primary diagnosis (untreated)

Abbreviations: CTL, comprehensive thyroid & lung; CUP, cancer of unknown primary; F, female; FISH, fluorescence in situ hybridization; FP, FusionPlex; IHC, immunohistochemistry; M, male; NOS, not otherwise specified; STUMP, Spitzoid Tumor of Uncertain Malignant Potential
